# Real-world efficacy and safety of transarterial chemoembolization plus sintilimab and bevacizumab biosimilar for intermediate-advanced hepatocellular carcinoma: a propensity score matching study

**DOI:** 10.3389/fimmu.2026.1748142

**Published:** 2026-04-10

**Authors:** Li-Wei Deng, Jun-Feng Liu, Hai-Yan Yu, Bo Peng, Bin Liu, Shi-Feng Feng, Dong-Xu Liu, Yan-Yuan Sun, Yan Chen, Jian-Jian Chen, Chi Zhang, Hai-Qing Wang, Guo-Hui Xu, Le Liu, Guo Wei, Yi Ren, Lei Cao, Yong Zhao, Xue-Gang Yang

**Affiliations:** 1 Department of Interventional Therapy, Sichuan Clinical Research Center for Cancer, Sichuan Cancer Hospital & Institute, Sichuan Cancer Center, Affiliated Cancer Hospital of University of Electronic Science and Technology of China, Chengdu, China; 2 Department of Oncology, Xuanhan County People’s Hospital, Dazhou, Sichuan, China; 3 Department of Interventional Radiology, Chengdu Second People’s Hospital, Chengdu, China; 4 Department of Tumor Vascular Intervention, Chengdu Qingbaijiang District People’s Hospital, Chengdu, China; 5 Department of General Surgery, Chengdu Public Health Clinical Medical Center, Chengdu, China; 6 Department of Pharmacy, Sichuan Clinical Research Center for Cancer, Sichuan Cancer Hospital & Institute, Sichuan Cancer Center, Affiliated Cancer Hospital of University of Electronic Science and Technology of China, Chengdu, China; 7 Department of Radiology, Sichuan Clinical Research Center for Cancer, Sichuan Cancer Hospital & Institute, Sichuan Cancer Center, Affiliated Cancer Hospital of University of Electronic Science and Technology of China, Chengdu, China; 8 Department of Hepatopancreatobiliary Surgery, Sichuan Clinical Research Center for Cancer, Sichuan Cancer Hospital & Institute, Sichuan Cancer Center, Affiliated Cancer Hospital of University of Electronic Science and Technology of China, Chengdu, China

**Keywords:** bevacizumab, chemoembolization, combined modality therapy, hepatocellular carcinoma, immunotherapy

## Abstract

**Objectives:**

This study aimed to investigate the efficacy and safety of transarterial chemoembolization (TACE) plus sintilimab and bevacizumab biosimilar as first-line therapy for intermediate-advanced hepatocellular carcinoma (HCC).

**Materials and methods:**

A total of 253 patients with HCC who received either TACE plus sintilimab and bevacizumab biosimilar (combination group, n=74) or TACE alone (monotherapy group, n=179) were included retrospectively. Propensity score matching (PSM) analysis was used to match patients. The objective response rate (ORR), progression-free survival (PFS), overall survival (OS), and safety of two groups were compared.

**Results:**

After propensity score matching (1:2), 65 patients in the combination group were matched to 100 patients in the monotherapy group. The ORR (63.1% vs. 40.0%, *p* = 0.004) was better in the combination group than those in monotherapy group. The combination group had higher median PFS (13.3 vs. 7.1 months; hazard ratio [HR] = 0.63, 95% confidence interval [CI], 0.41–0.87; *p* = 0.017) and OS (20.1 vs. 14.6 months; HR = 0.53, 95% CI, 0.35–0.81; *p* = 0.010) than those in the monotherapy group. Multivariate analysis confirmed that BCLC stage B, ECOG PS of 0, and combination therapy were associated with higher PFS and OS. Grade 3/4 TRAEs occurred in 21.5% of the patients in the combination group, and 14.0% of the patients in the monotherapy group.

**Conclusion:**

Compared to TACE monotherapy, TACE plus sintilimab and bevacizumab biosimilar showed significantly better ORR, PFS, and OS for intermediate-advanced HCC.

## Introduction

Hepatocellular carcinoma (HCC) is the most common cancer and a leading cause of cancer-related mortality worldwide (1). Although patients with early-stage disease may receive curative therapies by resection, liver transplantation, or ablation (2), approximately 70% of patients are diagnosed at intermediate-advanced stage of disease (Barcelona Clinic Liver Cancer [BCLC] stage B or C) and survival is poor (3–5).

Transarterial chemoembolization (TACE) is recommended by guidelines as first-line therapy for BCLC-B stage HCC (3, 6, 7), and it is also widely used in BCLC-C stage HCC in real-world practice (4, 8). However, in HCC managed with TACE alone, the median progression-free survival (PFS) is only 7–8 months and there is an urgent need to improve survival for patients with intermediate-advanced HCC (9).

The combinatorial strategy of immune checkpoint inhibitors (ICIs) and anti-vascular endothelial growth factor (anti-VEGF) antibodies has precipitated a paradigmatic transformation in the clinical management of unresectable hepatocellular carcinoma (uHCC). The IMbrave 150 study demonstrated that atezolizumab (a programmed death-ligand 1 [PD-L1] antibody) combined with bevacizumab (an anti-VEGF antibody), and the ORIENT-32 trial showed that sintilimab (a programmed death 1 [PD-1] antibody) combined with a bevacizumab biosimilar (an anti-VEGF antibody)—both as first-line therapies for uHCC significantly improve PFS and overall survival (OS) (10, 11).

Recently, the results from LEAP-012, EMERALD-1, and TALENTACE studies have demonstrated that TACE combined with ICIs (PD-1/PD-L1) and tyrosine kinase inhibitors (TKIs) or anti-VEGF antibodies improves PFS compared with TACE monotherapy in the first-line therapy of unresectable HCC (uHCC) (12–14). However, clinical studies are characterized by stringent eligibility criteria for patient enrollment, which entails rigorous screening of participants. It remains elusive whether patients with uHCC who are excluded from such clinical trials can derive therapeutic benefits from the combination regimen of TACE, ICIs, and anti-VEGF antibodies. Concurrently, there persists a paucity of long-term efficacy data regarding the application of TACE combined with sintilimab and bevacizumab biosimilar in the management of uHCC in a real-world setting.

In this multicenter, retrospective cohort study, we aimed to evaluate the efficacy and safety of TACE plus sintilimab and bevacizumab biosimilar for intermediate-advanced HCC in a real-world setting.

## Materials and methods

### Patients

This multicenter retrospective study included patients with HCC who received either TACE combined with sintilimab and bevacizumab biosimilar or TACE alone between January 2019 and June 2024 at four centers in China. Ethical approval was obtained from the Ethics Committees of the four participating centers: Sichuan Cancer Hospital (SCCHEC-02-2022-113), Chengdu Second People’s Hospital (KYPJ2024007), Chengdu Public Health Clinical Medical Center (KJ-K2024-62-01), and Chengdu Qingbaijiang District People’s Hospital (2021–06). Due to the retrospective nature of the study, informed consent was waived. HCC diagnosis was confirmed through histopathologically/cytologically or clinical criteria according to the guidelines of the American Association for the Study of Liver Diseases (AASLD) (6).

Inclusion criteria were: (1) patients received the combination therapy (i.e., TACE plus sintilimab and bevacizumab biosimilar) or TACE monotherapy as first-line therapy during the same period; (2) age ≥18 years; (3) BCLC stage B or C; (4) Child-Pugh class A or B; (5) at least one measurable intrahepatic lesion per modified RECIST (mRECIST) criteria (15); and (6) Eastern Cooperative Oncology Group (ECOG) performance status ≤ 1. The combination therapy was defined as administration of TACE before or after sintilimab and bevacizumab biosimilar within 30 days. At least one cycle of sintilimab and bevacizumab biosimilar should be used after the TACE procedure.

Exclusion criteria included: (1) prior systemic or locoregional therapy (hepatic resection, ablation, or radiotherapy) for HCC; (2) concurrent or previous malignancies other than HCC; and (3) incomplete data.

The treatment decision (TACE alone or TACE plus systemic agents) was based on individual circumstances and physician discretion. Multidisciplinary teams (MDT) for HCC at the participating hospitals made treatment decisions based on patients’ financial burden, potential treatment outcomes, and treatment-related complications.

Relevant clinical data of the patients were extracted from the electronic medical record systems of the participating centers.

### TACE treatment

In this study, patients received conventional TACE (cTACE) or drug-eluting beads TACE (DEB-TACE) that were performed by experienced interventional radiologists with more than 10 years of experience at each center, following established guidelines. Superselective catheterization and embolization techniques were employed using microcatheters (16). Detailed procedural protocols are provided in the **Supplementary Treatment protocol**.

Repeat TACE procedures were done “on demand” when residual viable tumor or intrahepatic recurrence was suspected based on follow-up contrast-enhanced computed tomography (CT) or magnetic resonance imaging (MRI). TACE was discontinued in cases of deterioration of liver function to Child-Pugh C, ECOG performance status > 2, or continuous progression of target lesions after three TACE sessions according to the clinical practice of the participating centers (17).

### Sintilimab and bevacizumab biosimilar administration

Patients received intravenous sintilimab (200 mg) and bevacizumab biosimilar (15 mg/kg) every 3 weeks in accordance with the ORIENT-32 trial (11). Temporary sintilimab and bevacizumab biosimilar was allowed because of toxicities. Dose reduction of bevacizumab biosimilar because of toxicities was allowed. Drugs were discontinued in the event of disease progression, unacceptable toxic effects, patient choice, or the recommendation of the physicians.

### Assessments and follow-up

Tumor response was evaluated by two independent radiologists with more than eight-year of experience based on follow-up contrast-enhanced CT or MRI. Tumor response was evaluated using the mRECIST. The senior radiologists made the final decision in case of any disagreement. Patients underwent regular follow-up evaluations every 4–12 weeks after initial treatment, including CT/MRI and laboratory tests.

Safety assessments were done continuously through laboratory tests and physical examination.

The treatment-related adverse events (TRAEs) were assessed according to the National Cancer Institute Common Terminology Criteria for Adverse Events (CTCAE; version 5.0). The final follow-up date was April 30, 2025.

### Outcome measurements

The objective response rate (ORR) was defined as the proportion of patients achieving complete response (CR) and partial response (PR). Disease control rate (DCR) was defined as the percentage of patients achieving the CR, PR, or stable disease (SD). PFS was defined as the interval from the date of enrollment time of two groups to the date of disease progression or death. OS was defined as the interval from the date of enrollment time to the date of death. For patients treated with systemic therapy (sintilimab and bevacizumab biosimilar) after TACE in the combination group or patients in the TACE monotherapy group, the enrollment time was defined as the date of the initial TACE procedure during the study period. For patients treated with systemic therapy before TACE, the enrollment time was defined as the date of initiation of systemic therapy.

### Statistical analysis

Statistical analyses were performed using SPSS version 25.0 (IBM Corp., Armonk, NY, USA) and R (version 4.1.0; R Project for Statistical Computing, http://www.r-project.org). To reduce the potential confounding and baseline imbalances between groups, propensity score matching (PSM) analysis was performed and 1:2 nearest-neighbor algorithm with a caliper width of 0.05 to balance the baseline characteristics, which included sex, age, BCLC stage, tumor distribution, Child-Pugh class, albumin-bilirubin grade, ECOG PS and Up-to-seven criteria. Student’s t-test or Mann–Whitney U test was used to analyze continuous variables which were expressed as median (interquartile range). Categorical variables were compared using chi-squared or Fisher’s exact tests. Survival curves were generated using the Kaplan-Meier method and compared via log-rank tests. Univariable and multivariable Cox proportional hazards models were used to evaluate the independent factors of survival based on the propensity-matched patients. The variables with *p* < 0.05 in univariate analysis were included in multivariable analysis. Statistical significance was set at *p* < 0.05 (two-tailed).

## Results

### Patient characteristics

**Figure 1:**
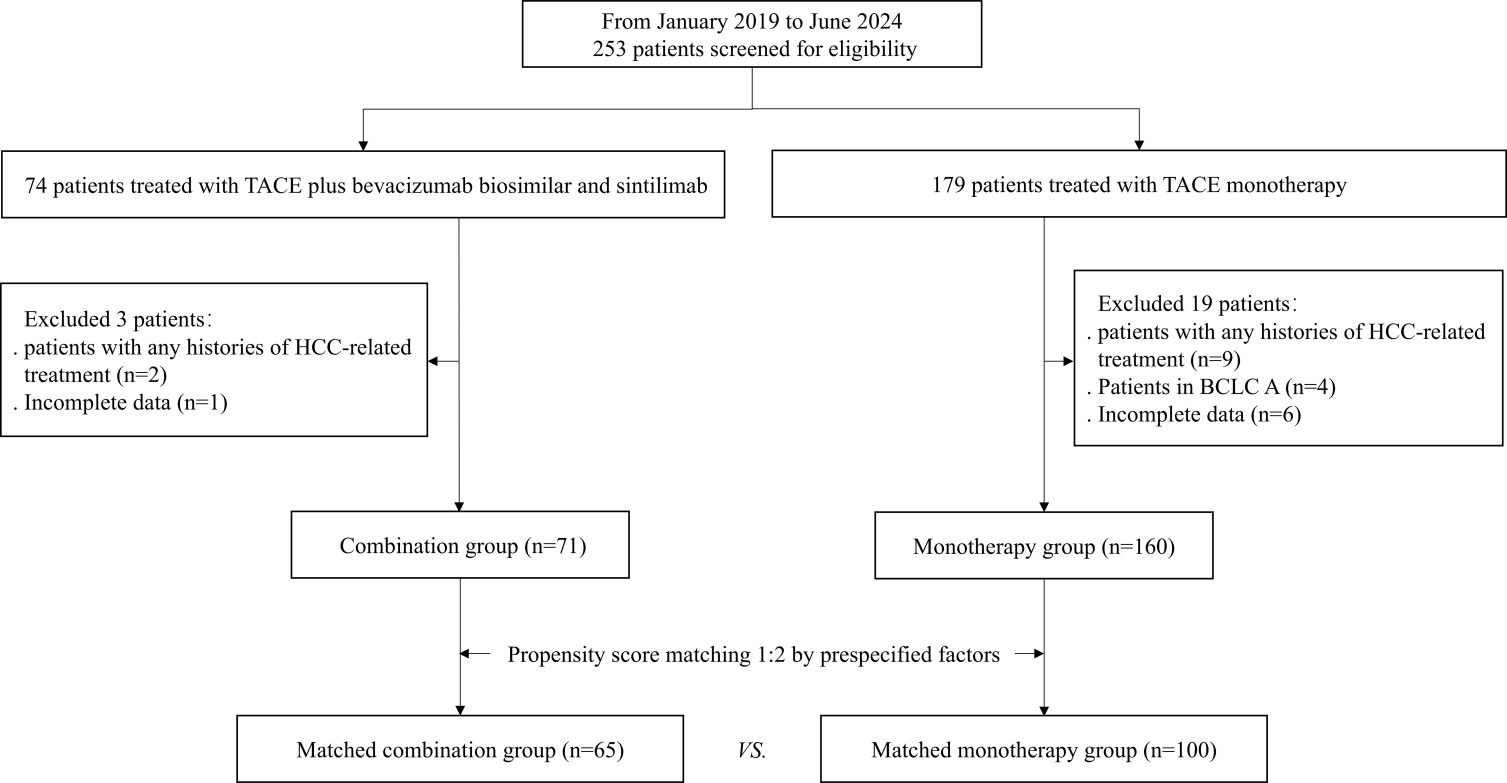
Patient selection flowchart. TACE, transarterial chemoembolization; BCLC, Barcelona Clinic Liver Cancer.

A total of 253 patients were included in this study, with 74 patients receiving TACE combined with sintilimab and bevacizumab biosimilar (combination group), and 179 patients receiving TACE alone (monotherapy group). After applying exclusion criteria, 71 patients were included in the combination group and 160 patients in the monotherapy group. Pre-PSM, more patients in the monotherapy group were over 60 years old than in the combination (*p* = 0.007). Post-PSM (1:2), 65 patients in the combination group and 100 patients in the monotherapy group. The patient selection flowchart was shown in **Figure 1**. Post-PSM, baseline characteristics including age, tumor distribution, and ALBI grade were well balanced with no significant differences (all *p* > 0.05, **Table 1**).

**Table 1:** Baseline characteristics of patients in the two groups before and after PSM.

Characteristics N (%)	Before PSM				After PSM			
	Combination group (n=71)	Monotherapy group (n=160)	*P* value	SMD	Combination group (n=65)	Monotherapy group (n=100)	*P* value	SMD
Age (years)	54.0 (50.0, 60.0)	57.0 (49.0, 67.0)	0.007	0.186	56.0 (50.0, 60.0)	54.5 (47.0, 60.0)	0.955	<0.001
< 60	54 (76.1)	92 (57.5)			49 (75.4)	75 (75.0)		
≥ 60	17 (23.9)	68 (42.5)			16 (24.6)	25 (25.0)		
Sex			0.242	0.058			0.960	0.015
Male	64 (90.1)	135 (84.4)			59 (90.8)	91 (91.0)		
Female	7 (9.9)	25 (15.6)			6 (9.2)	9 (9.0)		
Cirrhosis			0.686	0.025			0.455	0.023
Absent	20 (28.2)	41 (25.6)			19 (29.2)	24 (24.0)		
Present	51 (71.8)	119 (74.4)			46 (70.8)	76 (76.0)		
BCLC stage			0.850	0.013			0.850	0.061
B	28 (39.4)	61 (38.1)			25 (38.5)	37 (37.0)		
C	43 (60.6)	99 (61.9)			40 (61.5)	63 (63.0)		
Tumor size (cm)	8.3 (5.0, 10.8)	8.6 (5.3, 12.0)	0.542	0.038	8.5 (5.0, 10.9)	9.0 (6.1, 12.2)	0.404	0.031
< 5	20 (28.2)	39 (24.4)			18 (27.7)	22 (22.0)		
≥ 5	51 (71.8)	121 (75.6)			47 (72.3)	78 (78.0)		
Tumor distribution			0.060	0.132			0.435	0.046
Single lobe	36 (50.7)	60 (37.5)			30 (46.2)	40 (40.0)		
Multiple lobes	35 (49.3)	100 (62.5)			35 (53.8)	60 (60.0)		
Tumor number			0.746	0.023			0.339	0.100
≤ 3	29 (40.8)	69 (43.1)			25 (38.5)	46 (46.0)		
> 3	42 (59.2)	91 (56.9)			40 (61.5)	54 (54.0)		
Macroscopic portal vein invasion			0.475	0.050			0.499	0.015
Absent	40 (56.3)	82 (51.2)			36 (55.4)	50 (50.0)		
Present	31 (43.7)	78 (48.8)			29 (44.6)	50 (50.0)		
Vp1	6 (8.5)	14 (8.8)			4 (6.2)	10 (10.0)		
Vp2	9 (12.7)	29 (18.1)			9 (13.8)	19 (19.0)		
Vp3	13 (18.3)	26 (16.3)			13 (20.0)	15 (15.0)		
Vp4	3 (4.2)	9 (5.6)			3 (4.6)	6 (6.0)		
Extrahepatic spread			0.530	0.040			0.350	0.031
Absent	53 (74.6)	113 (70.6)			48 (73.8)	67 (67.0)		
Present	18 (25.4)	47 (29.4)			17 (26.2)	33 (33.0)		
Etiology			0.734	0.021			0.700	0.008
Hepatitis B virus	53 (74.6)	116 (72.5)			47 (72.3)	75 (75.0)		
Others	18 (25.4)	44 (27.5)			18 (27.7)	25 (25.0)		
Child-Pugh class			0.190	0.069			0.626	0.008
A	63 (88.7)	131 (81.9)			57 (87.7)	85 (85.0)		
B	8 (11.3)	29 (18.1)			8 (12.3)	15 (15.0)		
ALBI grade			0.084	0.130			0.480	0.023
1	23 (32.4)	31 (19.4)			18 (27.7)	24 (24.0)		
2	45 (63.4)	123 (76.9)			44 (67.7)	74 (74.0)		
3	3 (4.2)	6 (3.8)			3 (4.6)	2 (2.0)		
ECOG PS			0.354	0.065			0.515	0.015
0	45 (63.4)	91 (56.9)			41 (63.1)	58 (58.0)		
1	26 (36.6)	69 (43.1)			24 (36.9)	42 (42.0)		
Up-to-seven criteria			0.902	0.007			0.706	0.046
Within	12 (16.9)	26 (16.2)			9 (13.8)	16 (16.0)		
Beyond	59 (83.1)	134 (83.8)			56 (86.2)	84 (84.0)		
AFP (ng/mL)			0.921	0.007			0.969	0.015
< 400	36 (50.7)	80 (50.0)			31 (47.7)	48 (48.0)		
≥ 400	35 (49.3)	80 (50.0)			34 (52.3)	52 (52.0)		
TACE type			0.507	0.046			0.448	0.061
cTACE	45 (63.4)	94 (58.8)			39 (60.0)	54 (54.0)		
DEB-TACE	26 (36.6)	66 (41.2)			26 (40.0)	46 (46.0)		
TACE sessions			0.560	0.041			0.786	0.008
1-2	37 (52.1)	90 (56.2)			35 (53.8)	56 (56.0)		
≥ 3	34 (47.9)	70 (43.8)			30 (46.2)	44 (44.0)		

Data were median (interquartile range) or n (%). PSM, propensity score matching; SMD, standardized mean difference; BCLC, Barcelona Clinic Liver Cancer; ALBI, albumin-bilirubin; ECOG PS, Eastern Cooperative Oncology Group performance status; AFP, alpha-fetoprotein; TACE, transarterial chemoembolization; cTACE, conventional transarterial chemoembolization; DEB-TACE, drug-eluting beads transarterial chemoembolization.

### Objective response rate

Before PSM, patients in combination group had better ORR (66.2% vs. 40.0%, *p* < 0.001) and DCR (91.5% vs. 73.1%, *p* = 0.002) than those in the monotherapy group. After PSM, patients in the combination group also had better ORR (63.1% vs. 40.0%, *p* = 0.004) and DCR (90.8% vs. 73.0%, *p* = 0.005) than those in the monotherapy group. A summary of response rates in the two groups before and after PSM was provided in **Table 2**.

**Table 2:** Summary of response rates before and after PSM according to mRECIST.

Variable	Before PSM			After PSM		
	Combination group (n=71)	Monotherapy group (n=160)	*P* value	Combination group (n=65)	Monotherapy group (n=100)	*P* value
CR	12 (16.9)	11 (6.9)		11 (16.9)	6 (6.0)	
PR	35 (49.3)	53 (33.1)		30 (46.2)	34 (34.0)	
SD	18 (25.3)	53 (33.1)		18 (27.7)	33 (33.0)	
PD	6 (8.5)	43 (26.9)		6 (9.2)	27 (27.0)	
ORR	47 (66.2)	64 (40.0)	< 0.001	41 (63.1)	40 (40.0)	0.004
DCR	65 (91.5)	117 (73.1)	0.002	59 (90.8)	73 (73.0)	0.005

PSM, propensity score matching; CR, complete response; PR, partial response; SD, stable disease; PD, progressive disease; ORR, objective response rate; DCR, disease control rate.

### Survival

*Before PSM*, 69.0% (49/71) of patients in the combination group and 86.2% (138/160) of patients in the monotherapy group had disease progression or died. The median PFS and OS were significantly longer in the combination group than those in the monotherapy group (for median PFS, 13.3 months [95% CI, 8.9-17.7] vs. 7.5 months [95% CI, 5.2-9.6], *p* = 0.025; for median OS, 20.4 months [95% CI, 16.1-24.7] vs. 14.6 months [95% CI, 11.3-17.9], *p* = 0.007) (**Supplementary Figures 1A, B**).

**Figure 2:**
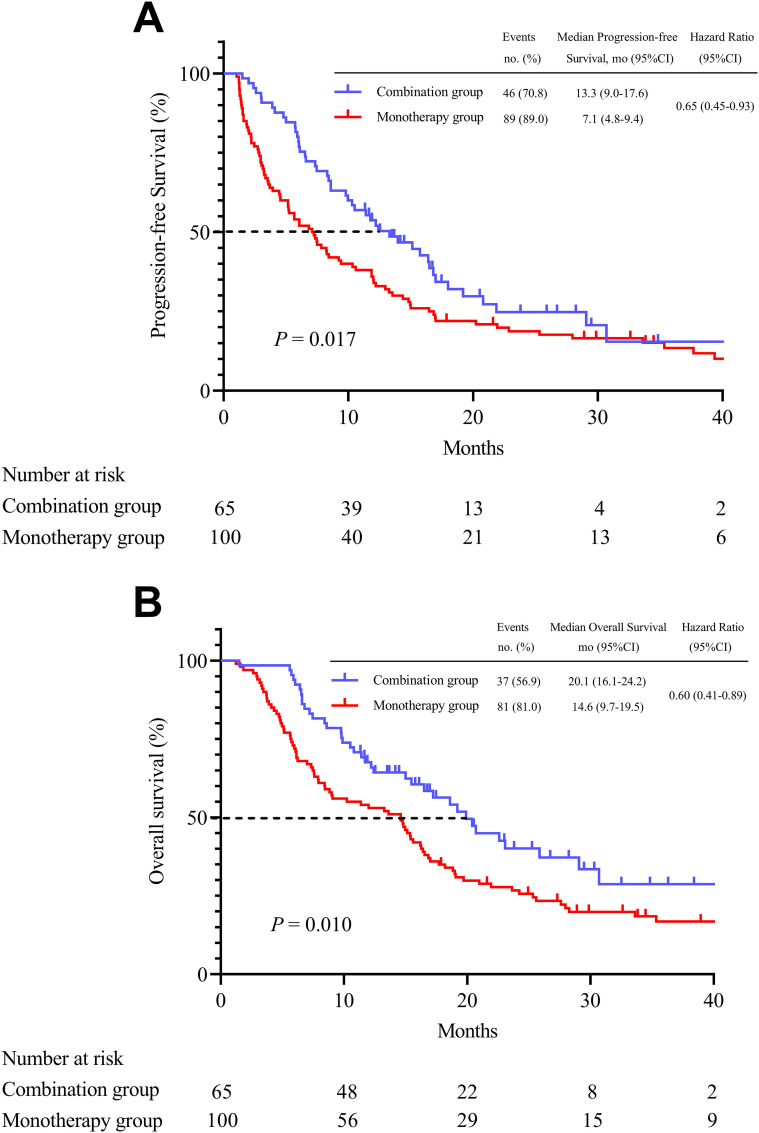
Kaplan-Meier analyses progression-free survival **(A)** and overall survival **(B)** according to two groups after matching.

**Figure 3:**
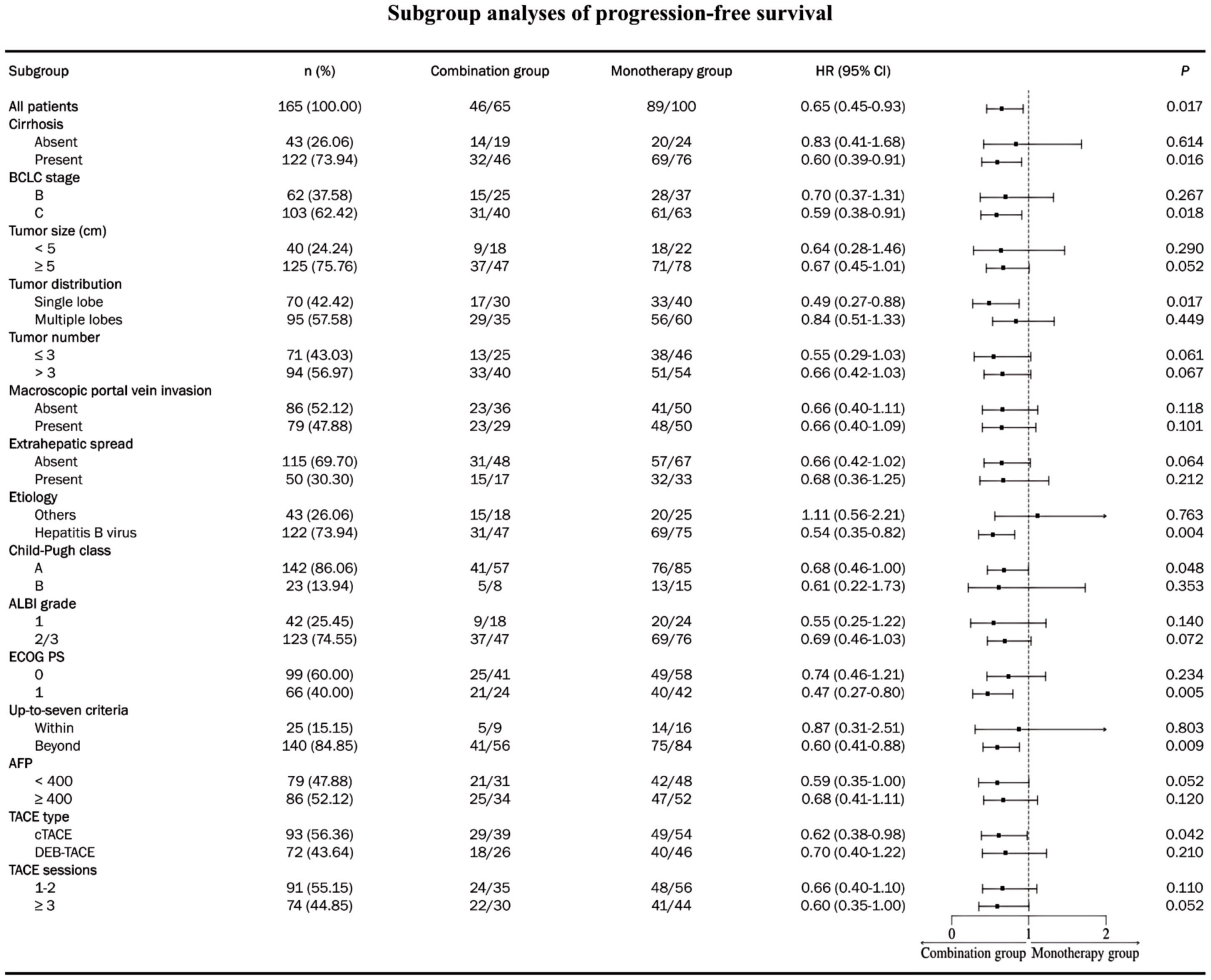
Subgroup analysis of progression-free survival after matching. HR, hazard ratio; CI, confidence interval; ECOG, Eastern Cooperative Oncology Group; BCLC, Barcelona Clinic Liver Cancer; TACE, transarterial chemoembolization; cTACE, conventional transarterial chemoembolization; DEB-TACE, drug-eluting beads trans arterial chemoembolization; HCC, hepatocellular carcinoma.

**Figure 4:**
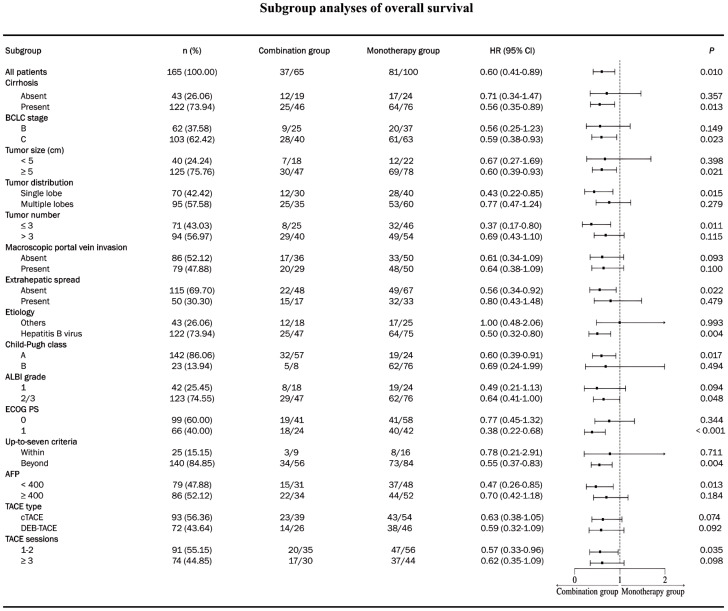
Subgroup analysis of overall survival after matching. HR, hazard ratio; CI, confidence interval; ECOG, Eastern Cooperative Oncology Group; BCLC, Barcelona Clinic Liver Cancer; TACE, transarterial chemoembolization; cTACE, conventional transarterial chemoembolization; DEB-TACE, drug-eluting beads trans arterial chemoembolization; HCC, hepatocellular carcinoma.

After PSM, five (7.7%) patients underwent conversion surgical resection after receiving combination therapy, compared with three (3.0%) patients in the monotherapy group. The median PFS and OS were significantly longer in the combination group than those in the monotherapy group (for median PFS, 13.3 months [95% CI, 9.0-17.6] vs. 7.1 months [95% CI, 4.8-9.4], *p* = 0.017; for median OS, 20.1 months [95% CI, 16.1-24.2] vs. 14.6 months [95% CI, 9.7-19.5], *p* = 0.010) (**Figures 2A, B**). Subgroup analysis revealed a consistent trend indicating longer benefits in PFS (**Figure 3**) and OS (**Figure 4**) with combination therapy than with monotherapy. Multivariate analysis confirmed that BCLC stage B, ECOG PS of 0, and combination therapy were associated with higher PFS and OS (**Table 3**).

**Table 3:** Univariate and multivariate predictors of progression-free survival and overall survival after PSM.

Variables	Univariate analysis			Multivariate analysis		
	HR	95% CI	*P* value	HR	95% CI	*P* value
PFS analyses						
Cirrhosis (Present vs. Absent)	1.17	0.79-1.73	0.425			
BCLC stage (C vs. B)	2.20	1.52-3.16	< 0.001	2.16	1.08-4.30	0.029
Tumor size (cm) (≥ 5 vs. < 5)	1.91	1.25-2.93	0.003	1.29	0.80-2.06	0.295
Tumor distribution (Multiple lobes vs. Single lobe)	1.54	1.08-2.18	0.017	1.21	0.80-1.82	0.373
Tumor number (> 3 vs. ≤ 3)	1.51	1.07-2.15	0.020	1.10	0.73-1.64	0.654
Macroscopic portal vein invasion (Present vs. Absent)	1.83	1.30-2.58	< 0.001	1.10	0.62-1.96	0.750
Extrahepatic spread (Present vs. Absent)	1.83	1.28-2.61	< 0.001	1.07	0.64-1.77	0.800
Etiology (Hepatitis B virus vs. Others)	1.24	0.84-1.82	0.280			
Child-Pugh class (B vs. A)	1.26	0.76-2.08	0.364			
ALBI grade (2 + 3 vs. 1)	1.50	0.99-2.26	0.055			
ECOG PS (1 vs. 0)	2.74	1.94-3.89	< 0.001	2.77	1.87-4.08	< 0.001
Up-to-seven criteria (Beyond vs. Within)	1.58	0.97-2.57	0.067			
AFP (ng/ml) (≥ 400 vs. < 400)	1.30	0.93-1.83	0.128			
TACE type (DEB-TACE vs. cTACE)	1.09	0.78-1.54	0.609			
TACE sessions (≥ 3 vs. 1-2)	1.02	0.73-1.44	0.890			
Treatment (Combination therapy vs. Monotherapy)	0.65	0.45-0.93	0.017	0.60	0.41-0.87	0.007
OS analyses						
Cirrhosis (Present vs. Absent)	1.10	0.74-1.67	0.609			
BCLC stage (C vs. B)	3.08	2.04-4.65	< 0.001	2.43	1.19-4.98	0.015
Tumor size (cm) (≥ 5 vs. < 5)	2.29	1.43-3.68	< 0.001	1.62	0.83-3.16	0.156
Tumor distribution (Multiple lobes vs. Single lobe)	1.76	1.21-2.56	0.003	1.27	0.82-1.99	0.289
Tumor number (> 3 vs. ≤ 3)	1.77	1.22-2.57	0.003	1.24	0.78-1.96	0.358
Macroscopic portal vein invasion (Present vs. Absent)	2.22	1.54-3.20	< 0.001	1.01	0.56-1.80	0.982
Extrahepatic spread (Present vs. Absent)	2.21	1.53-3.20	< 0.001	1.22	0.73-2.03	0.458
Etiology (Hepatitis B virus vs. Others)	1.29	0.86-1.94	0.212			
Child-Pugh class (B vs. A)	1.48	0.88-2.48	0.136			
ALBI grade (2 + 3 vs. 1)	1.32	0.86-2.03	0.201			
ECOG PS (1 vs. 0)	3.01	2.09-4.32	< 0.001	3.12	2.06-4.75	< 0.001
Up-to-seven criteria (Beyond vs. Within)	2.14	1.20-3.81	0.010	1.45	0.62-3.45	0.391
AFP (ng/ml) (≥ 400 vs. < 400)	1.42	0.99-2.04	0.058			
TACE type (DEB-TACE vs. cTACE)	1.21	0.84-1.74	0.306			
TACE sessions (≥ 3 vs. 1-2)	0.79	0.55-1.13	0.198			
Treatment (Combination therapy vs. Monotherapy)	0.60	0.41-0.89	0.010	0.53	0.35-0.81	0.003

PSM, propensity score matching; HR, hazard ratio; CI, confidence intervals; PFS, progression-free survival; BCLC, Barcelona Clinic Liver Cancer; ALBI, albumin-bilirubin; ECOG PS, Eastern Cooperative Oncology Group performance status; AFP, alpha-fetoprotein; TACE, transarterial chemoembolization; cTACE, conventional transarterial chemoembolization; DEB-TACE, drug-eluting beads transarterial chemoembolization; OS, overall survival.

### Safety

A total of 64.8% (107/165) of patients experienced any grade of TRAEs, with 67.7% (44/65) of patients in the combination therapy group and 63.0% (63/100) of patients in the TACE monotherapy group (**Table 4**). The most common TRAEs in the combination group were alanine aminotransferase (ALT) increase (29.2% [19/65] of patients with combination therapy vs. 18.0% [18/100] of patients with TACE monotherapy), aspartate aminotransferase (AST) increase (32.3% [21/65] vs. 25.0% [25/100]), and thrombocytopenia (15.4% [10/65] vs. 5.0% [5/100]). Grade 3/4 TRAEs occurred in 21.5% (14/65) of patients in the combination therapy group, and 14.0% (14/100) of patients in the monotherapy group (**Table 4**). No grade 5 TRAEs were observed in all patients. There were 6.1% (4/65) of patients with the discontinuation of sintilimab due to TRAEs, and 12.3% (8/65) of patients who suffered from dose reduction or interruption of bevacizumab.

**Table 4:** Treatment-related adverse events after PSM.

Events N (%)	Combination group (n=65)			Monotherapy group (n=100)		
	Any grade	Grade 1/2	Grade 3/4	Any grade	Grade 1/2	Grade 3/4
Any TRAE	44 (67.7)	41 (63.1)	14 (21.5)	63 (63.0)	61 (61.0)	14 (14.0)
Increased AST	21 (32.3)	15 (23.1)	6 (9.2)	25 (25.0)	20 (20.0)	5 (5.0)
Increased ALT	19 (29.2)	14 (21.5)	5 (7.7)	18 (18.0)	16 (16.0)	2 (2.0)
Hyperbilirubinemia	18 (27.7)	13 (20.0)	5 (7.7)	21 (21.0)	18 (18.0)	3 (3.0)
Pain	17 (26.2)	17 (26.2)	0 (0.0)	24 (24.0)	23 (23.0)	1 (1.0)
Decreased appetite	11 (16.9)	11 (16.9)	0 (0.0)	14 (14.0)	14 (14.0)	0 (0.0)
Nausea	11 (16.9)	11 (16.9)	0 (0.0)	12 (12.0)	12 (12.0)	0 (0.0)
Fever	10 (15.4)	10 (15.4)	0 (0.0)	11 (11.0)	10 (10.0)	1 (1.0)
Hypoalbuminemia	10 (15.4)	8 (12.3)	2 (3.1)	15 (15.0)	13 (13.0)	2 (2.0)
Thrombocytopenia	10 (15.4)	7 (10.8)	3 (4.6)	5 (5.0)	3 (3.0)	2 (2.0)
Neutropenia	8 (12.3)	7 (10.8)	1 (1.5)	6 (6.0)	5 (5.0)	1 (1.0)
Fatigue	7 (10.8)	7 (10.8)	0 (0.0)	8 (8.0)	8 (8.0)	0 (0.0)
Hypertension	7 (10.8)	5 (7.7)	2 (3.1)	12 (12.0)	11 (11.0)	1 (1.0)
Diarrhea	6 (9.2)	6 (9.2)	0 (0.0)	7 (7.0)	7 (7.0)	0 (0.0)
Hypothyroidism	6 (9.2)	6 (9.2)	0 (0.0)	0 (0.0)	0 (0.0)	0 (0.0)
Hand and foot syndrome	6 (9.2)	5 (7.7)	1 (1.5)	0 (0.0)	0 (0.0)	0 (0.0)
Gingival bleeding	5 (7.7)	5 (7.7)	0 (0.0)	0 (0.0)	0 (0.0)	0 (0.0)
Proteinuria	5 (7.7)	5 (7.7)	0 (0.0)	0 (0.0)	0 (0.0)	0 (0.0)
Gastrointestinal hemorrhage	3 (4.6)	2 (3.1)	1 (1.5)	1 (1.0)	1 (1.0)	0 (0.0)

PSM, propensity score matching; TRAEs, Treatment-Related Adverse Events; ALT, alanine transaminase; AST, aspartate aminotransferase.

## Discussion

This multicenter, retrospective cohort study of patients with intermediate-advanced HCC indicates that TACE plus sintilimab and bevacizumab biosimilar had a higher ORR, PFS, OS than TACE monotherapy, with manageable TRAEs. Multivariable analysis further confirmed combination therapy was an independent predictor for prolonged PFS and OS. The subgroup analyses showed a consistent trend indicating that combination therapy improved survival.

The CHANCE001 study demonstrated that TACE combined with ICIs and molecular targeted therapies significantly improved survival outcomes (PFS and OS) compared to TACE monotherapy (18). Notably, this study included a variety of PD-(L)1 inhibitors, anti-VEGF antibodies, and tyrosine kinase inhibitors (TKIs) from different companies (18). However, the current arbitrary combination of PD-(L)1 inhibitors with anti-VEGF antibodies or TKIs lacks consistency, which has hindered their clinical promotion and application. The TALENTACE study, which built on the IMbrave 150 study (atezolizumab plus bevacizumab) (10), evaluated TACE plus atezolizumab and bevacizumab as first-line therapy for uHCC (14). The study reported a median PFS of 10.3 months (95% CI, 7.5–15.0) and an ORR of 81.3% (14). Notably, this study included 2.0% (7/342) of patients with Vp1/2 portal vein tumor thrombus (PVTT) while excluding those with Vp3/4 PVTT and extrahepatic spread; meanwhile, the OS was not reached (14). Another prospective, single arm, phase 2 study demonstrated that TACE combined with intra-arterial infusion of sintilimab and bevacizumab for patients with advanced HCC achieved a median PFS of 6.0 months (95% CI, 4.8–not reached) and a median OS of 12.2 months (95% CI, 9.3–not reached) (19). Additionally, grade 3 and more TRAEs occurred in 11.7% (4/34) of patients (19). This study showed that TACE plus sintilimab and bevacizumab was safe and effective for HCC patients with Vp3/4 PVTT.

In our study, which built on the ORIENT-32 trial (sintilimab plus bevacizumab biosimilar) (11), evaluated TACE plus sintilimab and bevacizumab biosimilar as first-line therapy for intermediate-advanced HCC in a real-world setting. Our study included 25.5% (42/165) of patients with Vp1/2 PVTT, 22.4% (37/165) of patients with Vp3/4 PVTT, and 30.3% (50/165) of patients with extrahepatic spread. Meanwhile, the PFS and OS of the combination therapy were improved compared to those of TACE monotherapy. Our study comprehensively presents real-world data on the treatment of intermediate-to-advanced HCC with TACE plus PD-1 inhibitors and anti-VEGF antibodies, further filling some gaps in data from prospective clinical studies.

TACE embolizes the tumor-feeding arteries, leading to tumor cell necrosis. However, it concurrently triggers upregulation of VEGF and immunosuppression, both of which may contribute to disease progression (20, 21). Bevacizumab (anti-VEGF antibodies) normalizes aberrant tumor vasculature by suppressing VEGF-driven angiogenesis, alleviating hypoxia in the tumor microenvironment (TME) to enhance CD8^+^ T-cell infiltration and function, while simultaneously counteracting VEGF-mediated immunosuppression by restoring dendritic cell maturation and reducing regulatory T-cell (Treg) expansion (22–25). Meanwhile, PD-1/PD-L1 antibodies augment this immunomodulatory effect by blocking PD-1/PD-L1 interactions, revitalizing T-cell cytotoxicity, and upregulating interferon signaling pathways in T cells, natural killer (NK) cells, and macrophages. These actions further drive the secretion of proinflammatory cytokines and promote immune activation (23). These effects can convert an immunosuppressive “cold tumor” into an immunosupportive “hot tumor,” a transformation that boosts the immune response elicited by ICIs.

In this study, 21.5% (14/65) of patients in the combination therapy group and 14.0% (14/100) of patients in the monotherapy group experienced grade 3/4 TRAEs. The most frequent grade 3/4 TRAEs were increased AST (9.2% [6/65] of patients in the combination therapy group vs 5.0% [5/100] of patients in the monotherapy group). There were 6.1% (4/65) of patients with the discontinuation of sintilimab due to TRAEs, and 12.3% (8/65) of patients who suffered from dose reduction or interruption of bevacizumab biosimilar.

Our study has several limitations. First, as a retrospective study with small sample size, it may be susceptible to selection bias. To address this, we employed PSM to mitigate such potential biases, though residual confounding cannot be entirely ruled out. Second, over 70% of the patients in this cohort had HBV-related HCC. This raises questions about the generalizability of the combination therapy’s efficacy to patients with HCC of other etiologies, such as hepatitis C virus infection, non-alcoholic fatty liver disease, or alcoholic liver disease. Third, the real-world setting of this study introduced heterogeneity in TACE procedures across participating centers, which could influence treatment outcomes. Standardization of TACE protocols—including embolization materials, chemoagents used, and technical approaches—across centers may help reduce such variability in future studies (16).

In conclusion, compared to TACE monotherapy, TACE plus sintilimab and bevacizumab biosimilar provides significant ORR, PFS, and OS benefits for intermediate-advanced HCC patients, with manageable adverse events. The efficacy of this combination therapy still needs to be further validated in prospective, randomized trials.

## Data Availability

The raw data supporting the conclusions of this article will be made available by the authors, without undue reservation.
