# Supplementary material for: Real-world efficacy and safety of transarterial chemoembolization plus sintilimab and bevacizumab biosimilar for intermediate-advanced hepatocellular carcinoma: a propensity score matching study
Source: Front Immunol. 2026 Apr 10;17:1748142. doi: 10.3389/fimmu.2026.1748142 (PMC13106286; doi:10.3389/fimmu.2026.1748142)

**Treatment protocol**

**Transarterial chemoembolisation (TACE) procedure**

Hepatic artery angiography was performed with a 5-F RH catheter (Terumo) to assess the tumour location, number, size, and blood supply of target tumours. All the feeding arteries of tumours should be adequately obtained during the procedure, including vessels’ origin, variant anatomy, and ectopic or collateral blood supply. Subsequently, a microcatheter (Progreat; Terumo, Japan) was inserted into the feeding arteries of tumours.

Conventional TACE (cTACE) was an intra-arterial injection of 40-60 mg of epirubicin (Pharmorubicin; Pfizer, Wuxi, China) mixed with 5-15 mL of lipiodol (Jiangsu Hengrui Medicine Co., Ltd., Jiangsu, China). When needed, embosphere (100–300 μm or 300-500 μm) was used for further embolization to achieve stasis.

Drug-eluting bead TACE (DEB-TACE) was performed by CallSpheres® (Jiangsu Hengrui Medicine Co., Ltd.,Jiangsu, China) beads (100-300 μm) loaded with doxorubicin (40-60 mg) or HepaSpheres (Biosphere Medical, Inc., South Jordan, UT) with a diameter of 30-60 μm loaded with epirubicin (with a maximum dose of 50-80 mg). CalliSpheres® /HepaSpheres beads and non-ionic contrast agent were mixed by 1:1 and injected at a speed of 1 mL/min. The injection was completed during the stasis flow of contrast agent.

TACE was discontinued in cases of severe liver function (uncontrollable ascites, coagulation disorders, abnormal albumin, severe jaundice, overt hepatic encephalopathy), ECOG-PS > 2, and continuous progression of target lesions after 3 TACE sessions according to the clinical practice of every center.

**Supplementary Figure 1** The Kaplan-Meier curve of progression-free survival (A) and overall survival (B) before propensity score matching.


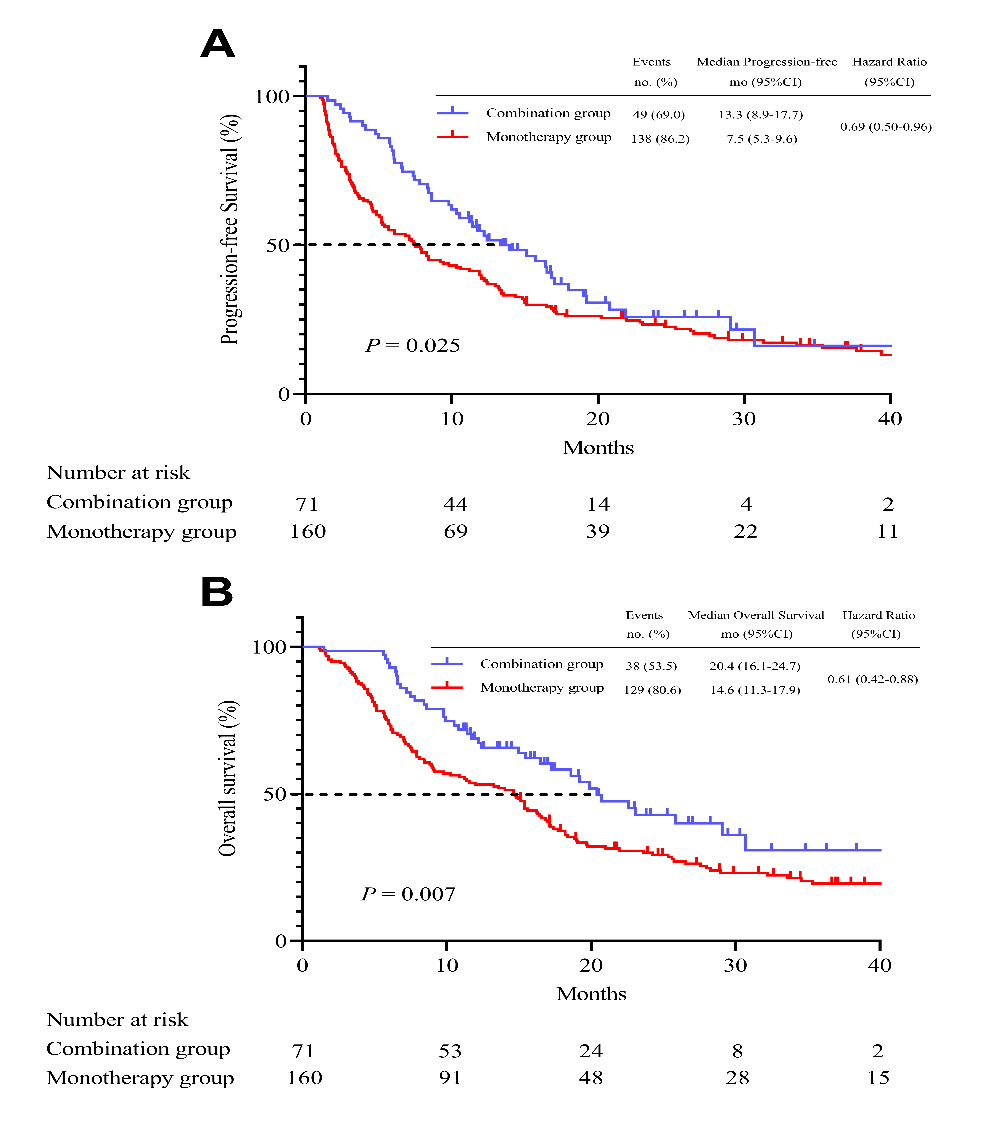

Supplement: Supplementary file 1 [file DataSheet1.docx]
